# Supraglottic airway use during neonatal resuscitation: better suited to aeration than medication?

**DOI:** 10.1038/s41390-025-04106-w

**Published:** 2025-04-29

**Authors:** Calum T. Roberts

**Affiliations:** 1 Department of Paediatrics, Monash University, Melbourne, VIC Australia; 2 The Ritchie Centre, Hudson Institute of Medical Research, Melbourne, VIC Australia; 3 Monash Newborn, Monash Children’s Hospital, Melbourne, VIC Australia

Supraglottic airways (SGAs), while more widely applied in other patient populations, have traditionally been something of an afterthought in neonatal care, with clinicians quicker to apply the ‘trusty’ face mask, or resort to endotracheal intubation. This status has started to change in recent years, as neonatal clinicians have increasingly acknowledged two important truths: 1. Face mask provision of positive pressure ventilation (PPV) is extremely hard to do well, with high rates of obstruction and leak,^1^ and 2. Intubation skills are declining, with reduced procedural opportunities and changing care guidelines, and outside of larger neonatal centers it may now be unrealistic to expect the clinicians performing neonatal resuscitation to be skilled intubators.^2^

The most recent review of SGA use during neonatal resuscitation conducted by the International Liaison Committee On Resuscitation (ILCOR) Neonatal Task Force showed resuscitation performed with SGA to have several advantages over face mask.^3^ The authors reported that data from 6 randomized trials (1823 infants) suggested that SGAs were associated with a reduced probability of failure to improve after PPV (relative risk [RR] 0.24, 95% confidence interval [CI] 0.17 to 0.36, moderate certainty) and a reduced probability of endotracheal intubation (RR 0.34, 95% CI 0.20 to 0.56, low certainty). These studies assessed resuscitation performed by clinicians with varying experience and skill sets, but notably all resuscitations in the largest trial (*n* = 1171) were performed by midwives.^4^ They underwent training immediately prior to the trial, which involved the need to perform three successful SGA insertions on a neonatal manikin. The clinical outcomes in this trial were similar in the SGA and face mask groups, but the findings are strongly suggestive that clinicians without advanced airway skills can establish competency in SGA use, and apply those skills in clinical practice, after only brief training. The most recent ILCOR treatment recommendation on SGA use during neonatal resuscitation suggested that, provided appropriate training and resources are in place, SGA can be used in place of face mask to provide PPV to infants born at 34 weeks’ gestation or above.^5^ Use in more preterm infants is limited by the size of currently available devices.

Given their effectiveness for PPV, it is natural to pose the question whether SGAs might be an effective route to administer medications. There is a growing body of work to suggest that provision of surfactant to infants with respiratory distress syndrome is feasible, and potentially effective, using an SGA.^6^ A recent Cochrane Review reported that SGA surfactant treatment may result in a reduced risk of mechanical ventilation versus the use of InSurE (intubation-surfactant-extubation), with RR 0.53 (95% CI 0.36 to 0.78), but cautioned that this was low certainty evidence based on relatively small studies.^7^ A larger multi-center RCT, comparing SGA surfactant treatment with minimally invasive surfactant treatment via thin catheter, is currently underway (ACTRN12620001184965).

In this issue of *Pediatric Research*, Ramsie and colleagues address SGA administration of another medication: epinephrine.^8^ Epinephrine use during neonatal resuscitation is a rare, but critical, intervention, reserved for the most severely compromised infants who have not improved after use of positive pressure ventilation and chest compressions. While it is estimated that epinephrine treatment is used at fewer than 0.1% of births, these infants are at particularly high risk of adverse outcomes, including death and disability.^9,10^ In the event that epinephrine is required, the ability to administer it rapidly presents an advantage, potentially reducing the time to return of effective spontaneous circulation, provided that the desired effect on the circulation is achieved. This may, in turn, produce better outcomes. SGA insertion is likely to be more quickly achievable than endotracheal tube (ETT) placement for many clinicians, with one study reporting that the SGA could be inserted within approximately half the time required (32 versus 66 s) to place an ETT.^11^

In this original preclinical work, Ramsie et al. focus on the effect of the epinephrine after administration, assessing the pharmacokinetic and pharmocodynamic effects of a 0.1 mg/kg dose, allocated randomly to be given by either SGA or ETT (tracheostomy). The treatment was provided to anaesthetized piglets, aged 1–3 days, who were clinically stable, rather than requiring resuscitation. The SGA piglets were further subdivided to receive the epinephrine via a catheter placed at either the proximal (‘SGA Top’ group) or distal end (‘SGA Bottom’ group) of the device, to determine whether this impacted the observed effect.

Pharmocokinetic assessment showed plasma epinephrine concentrations that did not statistically significantly differ in the three treatment groups, although the SGA Top group achieved the lowest values. The ETT epinephrine piglets were the only group to show a significant change from baseline in hemodynamic variables, including heart rate and cardiac output, seen within the first two minutes after administration. The SGA Top piglets showed little sign of any effect on the hemodynamic parameters assessed, and had a significantly lower stroke volume than the ETT group between 4 and 10 min after treatment. SGA Bottom piglets had values closer to the ETT group for variables such as cardiac output and stroke volume, but for most other assessed variables responded similarly to the SGA Top piglets.

There are of course some limitations to this study design: the piglets used had already effectively transitioned from fetal to newborn status with clearance of lung fluid, and anatomical differences between piglets and human infants may impact how the SGA performs. However, these findings do not suggest much promise for supraglottic airway epinephrine administration, especially given that, in a newborn with compromised circulation, drug absorption would be expected to be less effective than what is observed in the animals with intact circulation studied here. The authors speculate that the site of drug absorption, in part occurring at laryngeal mucosa rather than bronchoalveolar mucosa when the SGA is used, may explain some of the differences seen between groups.

It should be highlighted that the control group in this study, ETT epinephrine administration, is far from the ‘gold standard’. While ETT epinephrine administration remains an option in neonatal resuscitation guidelines, this is based on historical precedent rather than high quality evidence. Both preclinical studies, and observational cohorts of infants, have shown that administration of ETT epinephrine is frequently ineffective, and that subsequent intravenous doses are often required before circulation is restored.^12–15^ Clinical guidelines emphasize that intravenous administration (typically by umbilical venous cannula in the newborn) is the preferred route for epinephrine treatment.^16–18^ Intraosseous access may be used as an alternative,^16,18^ and has been shown to be similarly effective to intravenous epinephrine in preclinical trials, although clinical data are relatively few.^19^

Preclinical study findings suggest that ETT epinephrine may perform better in the setting of bradycardia and reduced cardiac output than for complete asystole.^13,20^ A recent publication reporting outcomes for infants included in a national resuscitation registry included 281 infants receiving a first dose of epinephrine via ETT.^21^ Fewer than half of these infants achieved return of circulation with ETT epinephrine only, with the remainder either responding after intravenous epinephrine, or not surviving. Acknowledging that there were some missing data, the response rate to ETT epinephrine alone (without subsequent intravenous epinephrine) was 64/118 infants (54.2%) initially assessed as bradycardic, and 31/82 infants (37.8%) assessed as asystolic.^22^ A preclinical study assessing a substantially higher dose of ETT epinephrine than the maximum clinical dose (1 mg/kg rather than 0.1 mg/kg) found that this resulted in a response rate closer to intravenous treatment.^13^ Such a higher dose could potentially improve response to SGA treatment, but the safety of this approach is unclear, and cannot be clinically recommended currently with either device. Other avenues to achieve a simpler and more generalizable approach epinephrine administration include the possibility of intranasal administration, which in animal studies also appears more promising in the setting of bradycardia than asystole, but is clinically untested.^14,20^

Neither the ETT route, which remains included in clinical guidelines (but only if intravenous access is not yet available),^16,18^ or the SGA route, which is purely experimental, appear to be the optimal approach to effectively administer epinephrine during neonatal resuscitation. At present, the SGA should be regarded as a highly effective device to establish an airway and provide respiratory support, which for most infants is sufficient to ensure effective stabilization. For the sickest infants requiring epinephrine treatment, intravenous access, or intraosseous as an effective alternative, should be the priority to provide the best chance of successful resuscitation.^16,18^
